# Behavior Analytic Feeding Interventions: Current State of the Literature

**DOI:** 10.1177/01454455221098118

**Published:** 2022-06-08

**Authors:** Keith Williams, Laura Seiverling

**Affiliations:** 1 Penn State Hershey Medical Center, PA, USA; 2 Ball State University, Muncie, IN, USA

**Keywords:** feeding problems, food refusal, food selectivity, tube weaning

## Abstract

The purpose of this paper was to review the current state of the behavior analytic feeding intervention literature. We highlight studies that we found to be important contributions to the recent literature in the following areas: food selectivity, chewing, packing, and food refusal/tube weaning and provide suggestions for future research and clinical work in these areas. We also discuss several current topics relevant to the field in hopes to further advance research and clinical practice. These topics include considering the benefits of innovative models of service delivery such as telehealth and caregiver-implemented interventions, the importance of evaluating long-term outcomes of behavioral feeding interventions, and lastly, ethical issues to consider in the designing and implementation of behavioral feeding interventions and training of practitioners in our field.

For over five decades, behavioral interventions have successfully treated a range of pediatric feeding problems. While food selectivity has been the predominant focus of these interventions over the years, interventions have grown in scope and complexity as they have been employed with an increasing variety of feeding problems (e.g., chewing difficulties, total food refusal, and packing). The goal of this review article is not to provide a review of recent feeding studies or to replicate the numerous existing literature reviews, but rather to review the current state of the behavioral feeding intervention literature to address a range of common pediatric feeding problems. We will highlight the shift to predominately antecedent-based intervention components to address food selectivity over the last several years and review recent interventions used to address selective eating in those diagnosed with avoidant restrictive food intake disorder (ARFID; American Psychiatric Association, 2013). We will also recognize the importance of recent studies comparing behavior analytic interventions to waitlist control or non-behavior analytic interventions to improve food selectivity. Next, we will provide an overview of recent advances in the chewing, packing, and food refusal/tube weaning intervention literatures and provide suggestions regarding future research and clinical practice in these areas.

In addition to reviewing what we have found to be important trends and contributions of the recent behavior analytic feeding literature, we will discuss some current topics relevant to the field in hopes to continue advancing research and clinical practice. These topics include the potential benefits of innovative models of service delivery such as telehealth and caregiver-implemented interventions, the importance of evaluating long-term outcomes of behavioral feeding interventions, and lastly, ethical issues to consider in the designing and implementation of behavioral feeding interventions and training of practitioners in our field.

## A Treatment Shift for Food Selectivity

Over the last several years, numerous studies have examined the effectiveness of behavioral feeding interventions to improve food selectivity in children. Recent research has focused on examining the effectiveness of interventions that are less intrusive alternatives to escape extinction. While the use of escape extinction to increase acceptance of food in children with food selectivity has a tremendous amount of empirical support, disadvantages include short-term increases in problem behavior and extinction-induced emotional and aggressive behavior (Bachmeyer, 2009) which can make such procedures undesirable in settings such as homes and schools. There has been a trend of recently published studies that examine solely antecedent-based interventions, such as modeling (Hillman, 2019; O’Connor et al., 2020), high probability response sequences (Silbaugh & Swinnea, 2019; Trejo & Fryling, 2018), and simultaneous presentation combined with stimulus fading (Cho & Sonoyama, 2020). Researchers have also examined differential reinforcement alone (de los Santos, & Silbaugh, 2020), and response shaping (Turner et al., 2020) to expand diet variety in children with food selectivity. The findings and contributions of these recent studies are summarized below.

### Modeling

Using a multiple baseline design across participants combined with a multiple probe design across foods, O’ Connor et al. (2020) evaluated the use of video modeling of contingencies alone and combined with direct exposure to the contingencies in the treatment of food selectivity of three boys, ages 5 through 12 years, with limited diet varieties. Treatment involved presenting videos in which models just consumed nonpreferred foods, models were exposed to differential reinforcement or differential reinforcement plus escape extinction was provided for acceptance of nonpreferred foods. Differential reinforcement was also provided for consumption of non-preferred foods for the participants. Researchers found that modeling alone increased consumption of one food for one participant while the video modeling combined with other treatment components increased consumption of some nonpreferred foods, but not others.

Hillman (2019) used a multiple baseline design to examine the effects of video modeling with and without reinforcement in the home setting on food selectivity in three children, ages 3 to 4 years, with autism spectrum disorder (ASD). Video modeling took place during dinner for all children. The video model involved a child consuming the target foods and making positive statements about the food. When reinforcement was included, both the models and participants were provided with bites of preferred food for eating the target foods. While video modeling alone increased acceptance of target foods by the participants, higher levels of food acceptance occurred for three participants once reinforcement was also provided.

The above studies nicely demonstrate the various ways modeling may be incorporated into food selectivity interventions, combined with and without other treatment components such as differential reinforcement for clients who have imitative repertoires and are able to attend to the modeling. Advances in technology have made it easier than ever to create and use video models as part of interventions. Until recently, food selectivity interventions have rarely included modeling as an intervention component. While modeling in its various forms alone may not increase acceptance of all novel foods, the results of these studies are promising. It is important for clinicians and researchers to consider the potential benefits of using this low cost, antecedent-based intervention based on observational learning to improve acceptance of new and non-preferred foods in children with food selectivity. This relatively easy-to-implement intervention strategy may be feasible to implement in a range of settings (e.g., home, clinic, and school) with individual clients or even groups of children who have food selectivity.

### High-Probability Instructional Sequence

Based on behavioral momentum, a high-probability instructional sequence is a behavioral intervention used to increase the probability that a learner will engage in a low-probability behavior (e.g., eating a low-preferred food) by presenting a series of high-probability demands the learner will likely perform before presenting the demand to engage in the low-probability behavior (Mace et al., 1988). Trejo and Fryling (2018) compared two variations of a high-probability instructional sequence using a combined multiple baseline across foods and alternating treatments design to improve consumption of low-preferred foods in a 9-year-old boy with ASD. In one condition, the high-probability instructional sequence was topographically similar to the low-probability task of food consumption (i.e., acceptance of water via spoon) while in the other condition, the high-probability instructional sequence was topographically dissimilar (i.e., participant asked to “touch head,” “clap hands,” or “give me five”). Both variations of the high-probability instructional sequence were effective in increasing consumption and decreasing inappropriate mealtime behavior for the client. On the contrary, Silbaugh and Swinnea (2019) failed to replicate the effects of the high probability instructional sequence in three children with ASD with food selectivity, ages 4 to 6 years. Thus, while previous studies have demonstrated positive effects of high-probability response sequence to improve acceptance of new and low-preferred foods in children with ASD (Meier et al., 2012; Patel et al., 2007), as with the antecedent-based intervention of modeling, it may not be effective as an intervention in isolation without additional treatment components for all children or even for an individual child across a range of target foods.

### Simultaneous Presentation Combined With Stimulus Fading

In a school setting, Cho and Sonoyama (2020) used a reversal design to evaluate the effectiveness of two interventions to improve acceptance of non-preferred foods in a 7-year-old boy with ASD. In the first intervention, researchers used stimulus fading of non-preferred foods into preferred foods while in the second intervention, they examined simultaneous presentation of a preferred food combined with stimulus fading in of non-preferred foods. While the first intervention did not show an increase in consumption of non-preferred foods, the participant’s consumption of the three target foods greatly increased with implementation of the second intervention. In line with the modeling and high-probability instructional sequence studies described above, interventions involving antecedent-based interventions such as stimulus fading and simultaneous presentation may not initially be successful; but in this study, additional antecedent-based modifications to the original intervention led to an increase in child acceptance of novel and non-preferred foods.

### Differential Reinforcement and Shaping

In a clinical case study, de los Santos and Silbaugh (2020) demonstrated that differential reinforcement with contingent access to preferred foods increased consumption of non-preferred foods in a 4-year-old boy with developmental delays and food selectivity. Initially, improvements in acceptance of target foods were not seen with the intervention in place, but after re-assessing client preference by conducting a formal preference assessment, a new edible reinforcer was identified. Thus, increasing the potency of the reinforcer without the need of a more intrusive procedure such as escape extinction was successful at increasing acceptance of target foods. While initial treatment sessions were implemented in a clinical setting, the intervention was successfully transitioned into the home setting. Further, clinicians were able to transition from the therapist feeding the child target foods to an arrangement in which an adult was not required to feed the child.

Turner et al. (2020) recently implemented a multiple baseline design across behaviors for two 6-year-old boys with ASD to evaluate the effects of a shaping procedure to increase acceptance of new foods across small and large food sets. The shaping procedure involved providing reinforcement for the following sequence of behaviors: touching, tasting, licking, putting food in the mouth, and lastly, eating the food. About 3 foods were presented using the shaping procedure when the small set of food was presented, and 15 foods were presented in the large food set. Measuring the percentage of correct behaviors and the cumulative number of foods with which the children interacted, researchers found that for one participant, the shaping procedure was effective across both large and small food sets, although the participant consumed many more foods in the large food set condition. For the other participant, the shaping procedure was successful at increasing some feeding behaviors (e.g., putting food into the mouth) with the large food set, but that consumption only occurred when the small food set was presented.

These studies provide additional ways that food selectivity interventions can be modified (e.g., re-assessing client preference or adjusting the amount of target foods being offered) to improve the effectiveness of the intervention) without resorting to more intrusive intervention components such as escape extinction.

### ARFID Interventions

Over the last several years, there have been a growing number of studies examining the effectiveness of behavioral and cognitive-behavioral interventions to expand diet variety in a range of patients diagnosed with ARFID (American Psychiatric Association, 2013). ARFID is a relatively new diagnosis in the DSM-5 Feeding and Eating Disorder section which involves selective eating patterns which can impact a child’s growth, nutrition, and psychosocial functioning. While there is a long-established research literature examining the effectiveness of behavioral feeding interventions to expand diet variety in those with food selectivity, very limited treatment research has been carried out specifically in the ARFID population. Recent studies have shown intensive multidisciplinary behavioral intervention (Volkert et al., 2021), home-based behavioral intervention (T. Taylor et al., 2019), and cognitive-behavioral treatment in a day treatment setting (Dumont et al., 2019) to be successful treatment modalities to expand diet variety in children and adolescents with ARFID. While these initial studies suggest behavioral interventions to address food selectivity are effective at treating selective eating in those with an ARFID diagnosis; more studies examining behavioral feeding interventions in various formats (e.g., weekly outpatient vs. day treatment) as well as studies comparing behavioral and cognitive-behavioral intervention for ARFID should be conducted.

### Innovative Comparison Studies

While behavior analytic feeding interventions have a vast amount of empirical support to treat feeding problems (Sharp et al., 2017), researchers have primarily used single subject research designs and not in a randomized control fashion to evaluate their effectiveness. In the first of its kind waitlist-controlled study, Peterson et al. (2020) evaluated its effectiveness to improve food selectivity in children with ASD. Peterson et al. (2020) were the first to randomly assign six children with ASD and food selectivity to either a behavior analytic intervention or waitlist control. Those receiving behavior analytic treatment were exposed to a multicomponent intervention to improve acceptance of 16 foods. The waitlist group was subsequently exposed to the intervention. Percentage of acceptance of novel foods increased in the behavior analytic intervention group but not for the waitlist control until they were exposed to the intervention.

Despite the amount of empirical support for behavioral feeding interventions, many clinicians still opt to use non-behavior analytic approaches, such as sensory integration and sensory-based approaches (Schaaf & Miller, 2005), to treat food selectivity and other pediatric feeding disorders. In 2016, Peterson et al. compared the effectiveness of a sensory-based and behavior analytic approach to treat food selectivity and found the behavioral intervention to be more effective at increasing consumption of target foods. In a more recent study, Seiverling et al. (2018) used an alternating treatments design to compare a behavior analytic intervention with and without sensory integration therapy to improve food selectivity in two boys, ages 5 and 6 years, with ASD and food selectivity. During the sensory integration therapy condition, a series of sensory integration activities were implemented prior to the start of the meal in which the behavioral feeding intervention was implemented. Both participants showed similar increases in bites, drinks, and total amount consumed with decreases in inappropriate mealtime behavior in both conditions. Thus, caregivers were subsequently trained to implement the behavioral feeding intervention without sensory integration therapy.

Future researchers should consider branching out from traditional single subject research designs through randomized control studies with larger groups of participants to evaluate the effectiveness of behavior analytic feeding interventions to address food selectivity as well as the wider range of pediatric feeding disorders. More studies comparing behavior analytic and non-behavior analytic feeding interventions are also needed as there remains a paucity of studies comparing behavior analytic to other treatment approaches.

## Teaching Chewing

While the majority of behavior analytic feeding interventions over the years have focused on expanding diet variety in children with ASD who exhibit food selectivity, a portion of children with pediatric feeding problems, especially those with neuromuscular disorders (e.g., Down Syndrome and cerebral palsy) and craniofacial anomalies are likely to have oral motor skill deficits that impact eating and will need to be addressed as part of feeding intervention (Field et al., 2003; Selim, 2016). There is a small but growing behavior analytic literature that focuses on teaching the skill of chewing. Two recent studies have built upon previous studies by examining a novel treatment package to target the skill (T. Taylor, 2020) and targeting the individual skill of tongue lateralization as part of chewing instruction (Adams et al., 2020). T. Taylor (2020) recently used a modified multiple baseline probe across food textures to examine a multi-component intervention to increase food texture and chewing in a 4-year-old diagnosed with ASD and ARFID with no history of chewing. Intervention involved the use of contingent access to a preferred tablet and differential attention for chewing, non-removal of the bite presentation (with between side teeth placement) if refusal behavior occurred, re-presentation for expulsion, feeder modeling of tongue lateralization, and re-distribution of food with a coated baby spoon to move food in between the opposite side teeth if no chewing occurred or if the food was not on the child’s side teeth. At the end of the intervention, the participant showed an increase in chews per minute across a range of food textures.

Adams et al. (2020) used a multiple treatment design with probes to examine improvements in both tongue lateralization and chewing in one typically developing 4-year-old boy, an 8-year-old boy with ASD, and a 7-year-old female with an intellectual disability and ASD who had previously undergone craniofacial surgery to repair an anomaly of the lower jaw and cleft palate. All participants consumed purees and had no history of chewing prior to intervention. The intervention involved differential reinforcement (i.e., praise and preferred items provided contingent upon performing target skills), therapist modeling of both chewing and laterization, and verbal prompting. The skills of lateralizing and biting were initially targeted in separate sessions and were then combined into the same treatment sessions. An exit criterion component for biting was added to the intervention for one participant due to his refusal to bite down on the pieces of food presented. The exit criterion involved having the therapist present the bite of food by holding it on his molars until he bit down one time before terminating the session. Following intervention, all participants showed improvements in percentage of bite presentations with both chewing and tongue lateralization and transitioned to eating table foods.

These recent studies nicely contribute to the chewing literature as it is important for clinicians to consider the individual skills (e.g., tongue lateralizing and biting down) that may need to be taught to master this complex skill. Operational definitions are also becoming more refined as chewing can be a very challenging behavior to measure.

## Reducing Packing

Packing has been defined as not swallowing food or liquid within a specific duration, often 30 seconds. It has been defined functionally as an escape behavior which children exhibit to avoid swallowing food or liquid (Sevin et al., 2002), possibly because the food or liquid has been associated with gagging or choking or because the food is novel or non-preferred. It has also been suggested children pack or hold food in their mouths when they lack the skills to chew or swallow efficiently (Patel et al., 2005). In examining the studies included in a review of the packing literature (Silbaugh et al., 2018), we noted participants described had identified oral motor dysfunction or conditions often associated with oral motor dysfunction. These participants included children with vocal cord paralysis, delayed esophageal clearance, low facial muscle tone, chromosomal anomaly, and septo-optic dysplasia.

While literature describing interventions for packing has continued to grow, there has been only a single examination of the prevalence of packing among children with feeding problems. In a record review of 225 children treated in an intensive pediatric feeding program, 63% exhibited packing, with 42% exhibiting clinically high levels of packing (Rivero & Borrero, 2020). When defined more broadly as holding food/liquid in the mouth for prolonged periods of time, researchers have described packing in samples of children with CHARGE association (Hudson et al., 2016), Down Syndrome (Gisel et al., 1984), and cerebral palsy (Reilly et al., 1996). While prevalence studies are scarce, it is probable that packing is common among children with oral motor dysfunction.

### Redistribution

In one of the earlier studies to address packing, clinicians used a series of interventions to address the avoidance behaviors of a preschooler who refused most solid foods (Sevin et al., 2002). Clinicians used nonremoval of the spoon or presenting the spoon of food to the lips until acceptance, to increase acceptance while ignoring disruptive behavior. When the child’s expulsion food increased, clinicians used re-presentation, or placing expelled food back into her mouth, to decrease expulsion. When the child exhibited increased packing, redistribution, or collecting the food from her cheek pockets or under her tongue with a soft rubber-tipped brush and placing it on the center of her tongue every 15 seconds until she swallowed, was implemented. These authors suggested the behavioral mechanisms responsible for the treatment effects were either escape extinction or positive punishment (Sevin et al., 2002). We examined 15 studies on packing, including those from the Silbaugh review and 8 published after the review. Redistribution was included in 6 of 15 studies published subsequent to Sevin et al.’s (2002) study, making it the most researched intervention for packing. In the most recent study, researchers compared two redistribution interventions, in one intervention, clinicians implemented redistribution at both 15 and 30 seconds and in the other intervention, clinicians implement redistribution only after 30 seconds (Bloomfield et al., 2021). They found implementing redistribution more often resulted in greater reduction in packing. The authors stated “In our clinic’s typical treatment progression, we often first conduct an assessment comparing methods to place the bolus on the tongue (e.g., upright to flipped-spoon or Nuk presentation) if packing emerges. If packing persists, we then add redistribution” (Bloomfield et al., 2021, p. 1).

### Use of a Chaser

While the use of redistribution as a treatment of packing is most frequently documented in the literature, we predict the most widely used clinical intervention for packing is the use of a chaser. A chaser, often in the form of a small amount of liquid or low texture solid food, is presented after a bite of a target food that the child frequently packs (Williams & Foxx, 2007). Vaz et al. (2012) demonstrated the effectiveness of a liquid chaser for two children and a pureed food chaser for a third child. They noted the chaser was effective and arguably less intrusive than redistribution. Currently, it is not clear whether a chaser is as effective as redistribution for the treatment of packing. One study found that while a chaser reduced packing of purees to low levels in a young boy with ASD, it was insufficient to reduce the frequency of packing of table foods without the addition of redistribution (Levin et al., 2014).

### Simultaneous Presentation

Simultaneous presentation, discussed earlier as a treatment for food selectivity, has been used in two studies to decrease packing. In the first study, target foods were presented with small amounts of chocolate cookie (Buckley & Newchok, 2005) to reduce packing in a 9-year-old girl with ASD who packed non-preferred foods. In a more recent study, chocolate chips were presented with target foods (Whipple et al., 2020) to address packing of non-preferred foods in a 4-year-old boy with ASD. A contribution of the most recent intervention was that researchers were able to systematically fade out the simultaneous presentation across the course of the intervention with the child maintaining low levels of packing behavior. In both simultaneous presentation and use of a chaser, a preferred food or liquid facilitates swallowing of the target food. For both the chaser and simultaneous presentation it is a requirement for the child to have preferred foods. For children completely dependent on tube feeding who have little history of eating may resist the chaser as much as the target foods.

### A “Move On” Treatment Component to Address Packing

T. Taylor (2021) recently evaluated what was described as a “move-on” component to increase consumption and reduce packing in a 5-year-old girl with ASD with ARFID who had food selectivity, growth impairment, and exhibited packing behavior as well as overstuffing and vomiting during meals. Prior to the intervention, an evaluation revealed that the child was able to chew and swallow and thus, the packing behavior was not deemed to be the result of oral motor deficits. The intervention involved multiple components including differential reinforcement, re-presentation of expelled bites, and escape extinction. When the “move on” component was evaluated, it involved moving on to the next bite after 30 seconds instead of requiring a clean mouth before presenting the next bite (with a maximum of four bites presented as part of the procedure). With the “move on” component added, latency to clean mouth (i.e., no food or liquid in the mouth after 30 seconds) decreased and consumption increased to 100%. Additional treatment procedures of interspersing drink presentations and rotating different food textures as well as re-distribution were subsequently added as larger food portions were presented.

### Packing Prevention

A pair of studies provided a possible alternative to interventions for packing through the prevention of packing. In one study, clinicians offered three children with poor oral intake different food textures in a systematic assessment that revealed the children were able to eat lower textures foods with little to no packing (Patel et al., 2005). The parents of two of the three children used texture fading to gradually increase the texture of the foods their children consumed to regular texture table foods over the course of several months. Another study expanded on Patel et al.’s (2005) methodology by including the assessment of both texture and food type in the treatment of a girl dependent upon gastrostomy tube feeds (Kadey et al., 2013). While these authors also found lower texture was related to increased mouth clean, they further found the participant was more likely to pack some foods than others. Finally, the authors lowered the texture of the foods most likely to be packed and found decreasing texture reduced packing (Kadey et al., 2013).

While effective, redistribution will probably not be implemented widely outside of behaviorally-oriented feeding programs. The use of a chaser and simultaneous presentation have both been shown to be effective in a few studies, however, as mentioned, the child would need to have preferred liquids and solids in their repertoire in order for these interventions to be successful. While the “move on” procedure implemented by T. Taylor (2021) was shown to be effective, additional treatment components such as re-distribution were still subsequently added in order to maintain treatment gains. As shown by Patel et al. (2005) and Kadey et al. (2013), another option would be to prevent packing through changing texture or bite size, then using fading, probably coupled with reinforcement, to increase consumption of foods that the children have a history of packing. Patel et al.’s (2005) observation that their participants did eventually develop the skills necessary to eat regular texture food was important as it provides a possible framework for interventions used when packing behavior may be attributed to oral motor skill deficits.

## Food Refusal/Tube Weaning

Over a decade ago, we reviewed the intervention literature for food refusal, which we defined as a child’s refusal to eat all or most foods presented, resulting in the child’s failure to meet his or her calorie or nutritional needs (Williams et al., 2010). In this review, we examined 38 studies which included 218 children, 87% of whom received supplemental tube feedings at the beginning of the studies. The majority (61%) of studies included some form of escape extinction and a minority (11%) reported attempting to manipulate appetite. In a more recent review of feeding interventions used with children with ASD, Ledford et al. (2018) reported acceptance of food was addressed with nonremoval of the spoon in 43% of studies and scheduling or restricting food or liquid in 9% of studies. Although not specific to food refusal, Ledford et al.’s (2018) review reflected our field’s preference for the use of extinction-based interventions and limited usage of treatment components designed to provoke hunger.

In our review of the food refusal literature, 190 of 218 participants from the studies included received tube feedings (Williams et al., 2010). While tube feeding is common among the participants in studies on food refusal, there is a related, yet largely separate literature on tube weaning. Numerous studies describe tube weaning interventions in which appetite manipulation is the primary treatment component. Some of these studies involve behavioral components (e.g., Byars et al., 2003), while others do not (e.g., Gardiner et al., 2017). There is an interesting difference in perspective between the food refusal and tube weaning literatures. In the food refusal literature, interventions focus on both increasing behaviors that will promote oral intake, namely acceptance and swallowing of food, while decreasing behaviors interfering with intake, such as food expulsion or holding. Changes in these target behaviors allow for the elimination of tube feeds. In the tube weaning literature, changes in these target behaviors over time are seldom, often never, mentioned. The focus of many tube weaning studies is the reduction of the tube feeds to promote hunger which in turn results in the child’s oral intake increasing to the point of remaining off tube feeds.

A recent review found several studies used primarily or exclusively appetite manipulation for tube weaning. In this review, researchers identified 46 studies (26 single-subject, 19 group) single-subject) which they categorized by methodology, namely, behavioral, appetite manipulation, or a combination of these approaches (S. A. Taylor et al., 2019). Of the 26 single-subject studies, all used behavioral interventions. Of the 19 group interventions, 7 used behavioral interventions, 5 used appetite manipulation, and 7 used a combination of the two approaches. While the frequency of children weaned completely from tube feeds was, on average, highest with appetite manipulation, there were three key differences in participant characteristics between the approaches. The children in the appetite manipulation studies were, on average, younger, less likely to have special needs, and more likely to be dependent upon nasogastric tubes rather than gastrostomy tubes. Younger children probably have less well-established repertoires of avoidance behaviors than older children. Children with special needs are more likely to have both oral motor deficits and medical problems than children without special needs (Field et al., 2003; Williams et al., 2005), which may affect the intensity or duration of intervention. As gastrostomy tubes are generally recommended when non-oral nutrition will last longer than 6 weeks (Homan et al., 2021), children with nasogastric tube feeds probably have a shorter duration of tube dependency than children with gastrostomy tubes. Given differences in participant characteristics, it is not possible to conclude appetite manipulation is more effective than either a behavioral or combined approach, but this review suggests appetite manipulation is a successful approach to tube weaning. A comparison of two non-behavioral studies published after S. A. Taylor’s review also suggest the benefit of appetite manipulation. Bandstra et al. (2020) described their outcomes with 47 tube dependent children treated in an intensive program. They reported 40% of the children were completely weaned at the end of treatment with 36 days as the average length of treatment. They further reported appetite manipulation was not part of intensive treatment and changes to tube feeds were contingent upon oral intake (Bandstra et al., 2020). Another study described the outcomes of an intensive program in which a 65% tube reduction was made at the beginning of treatment (Kim et al., 2021). Kim et al. (2021) reported 81% of children were weaned at the end of a 19-day course of treatment. In the program with appetite manipulation the clinical course of treatment was briefer and the percentage of children weaned from tubes was higher. Again, direct comparison is problematic, but it again points to the effectiveness of appetite manipulation.

While there is evidence to support the use of appetite manipulation for tube weaning, we have little information on how interventions using this approach change behavior. The studies using exclusively appetite manipulation typically lack data on oral intake. They also do not include data on other variables of clinical interest such as expulsion of food, holding of food, and inappropriate mealtime behavior, and how these variables change across the span of treatment. In many studies describing behavioral treatments of tube weaning or food refusal, clinicians employ extinction procedures to address a range of behaviors such as turning away from the spoon, expelling the food, or refusal to swallow the food. It is unknown how appetite manipulation would affect the frequency and severity of the avoidance behaviors (e.g., turning away from the spoon, expulsion, and packing) so often reported in our literature. We would predict that combining appetite manipulation and behavioral interventions would reduce the need for extinction-based procedures. This said, it is important to recognize the induction of hunger takes time. In a description of their appetite-manipulation based treatment of tube weaning, Nowak-Cooperman and Quinn-Shea (2013) reported “it appears that it takes an average of 7 days of decreased calories before a child shows any significant increased eating.” These authors also describe reduction of tube feeds prior to the beginning of their 2-week intensive intervention. Pre-intervention appetite manipulation would seem to be an efficient way increasing hunger to maximize treatment effects but this has not been examined to date.

Although several clinicians have described an integration of appetite manipulation into their behavioral interventions (Brown et al., 2014; Byars et al., 2003), appetite manipulation is not widely described by behaviorally oriented clinicians. We expect, however, many clinicians utilize appetite manipulation, especially among children who are not tube dependent. Placing children on meal and snack schedules, eliminating grazing, and reducing drinking free access to milk or formula across the day are all forms of appetite manipulation. It is understandable that clinicians and researchers do not report appetite manipulation as a treatment component, as the effects of hunger are difficult to measure and systematically manipulate. Despite the potential difficulty of examining the effects of appetite manipulation, this is clearly an area which would benefit from further examination. There are numerous studies which use appetite manipulation and no escape extinction-based treatment components whose outcomes are at least equal to studies using extinction. At the very least, we need to determine how we can use appetite manipulation to reduce or eliminate the need to escape extinction-based procedures to treat food refusal and other feeding problems.

Table 1 highlights some of the advances in the recent literature for the range of feeding problems discussed above. In addition, we’ve summarized some of our suggestions for clinicians and future researchers as they continue their clinical work and research in the areas of food selectivity, chewing, packing, and food refusal/tube weaning.

**Table 1.** Recent Advances in the Behavior Analytic Feeding Literature and Suggestions for Clinicians and Future Researchers.

Feeding Problem	Recent Advances in the literature	Suggestions for clinicians and future researchers
Food Selectivity	Increasing empirical support for antecedent-based interventions and alternatives to escape extinction More studies being conducted to treat selective eating in those with ARFID Recent comparison studies add to empirical support for behavior analytic feeding interventions.	1. Consider the potential benefits of observational learning strategies as part of food selectivity interventions. 2. Explore the use of antecedent-based interventions such as high-probability instructional sequence, simultaneous presentation, and stimulus fading on acceptance of novel foods. 3. Further examine shaping procedures and differential reinforcement to increase acceptance of novel foods without escape extinction. 4. Determine the effects of behavior analytic feeding interventions in various formats (e.g., weekly outpatient vs. day treatment) to treat ARFID and compare behavioral interventions to cognitive-behavioral interventions for ARFID. 5. Continue comparison studies which examine the effectiveness of behavior analytic and non-behavior analytic interventions. Consider branching out from traditional single-subject research designs.
Chewing	Recent studies have used novel interventions to teach the skill	1. It is important to consider the individual skills (e.g., tongue lateralizing, biting down) that may need to be targeted in order to teach this complex skill.
Packing	A range of interventions to address packing have been documented in the literature (e.g., redistribution, a chaser, texture fading).	1. In order to determine the type of intervention to use, one must consider the client’s oral motor skills and level of food refusal with familiar foods and liquids. 2. More studies directly comparing different packing interventions should be conducted.
Food refusal/tube weaning	Recent studies have shown interventions that use primarily appetite manipulation, behavioral intervention combined with appetite manipulation, and behavioral interventions without appetite manipulation to successfully reduce tube feeds	1. Studies comparing these various approaches to tube weaning should be conducted. 2. Client characteristics must be considered when developing a tube weaning intervention. 3. Clinicians should be mindful of the role of appetite when designing behavior analytic feeding interventions. The use of appetite manipulation may reduce the need for more intrusive intervention components such as escape extinction.

## Current Topics to Consider for Advancement of Our Field

In addition to highlighting how the field of behavior analytic feeding interventions to treat a range of feeding problems has evolved over the last few years and providing suggestions for future researchers, we wanted to address several important topics that we feel are crucial to consider as we strive to continue improving the quality of both treatment and outcomes of behavior analytic feeding interventions.

### Innovative Service Delivery Models

Feeding programs consisting of multidisciplinary teams and a dedicated treatment space has been a major advance as these programs have allowed behavioral providers to implement interventions with the support of colleagues with expertise in medicine, nutrition, and oral motor functioning. As late as the 1980s, one could count the number of feeding programs in the United States on a single hand. Now a glance at the Feeding Matters website (www.feedingmatters.org) reveals dozens of multidisciplinary feeding programs nationwide. While there is considerable variability among feeding programs in terms of composition of their treatment teams, type of feeding problems addressed, and their approach to treatment, many, perhaps most, include a behavioral provider in the form of a behavior analyst or psychologist.

### Intensive Multidisciplinary Feeding Programs

Intensive treatment programs, both inpatient and day treatment have been the primary proving grounds for our field for the development and refinement of treatments. These treatments then find their way to an array of settings where they are implemented by a range of providers or caregivers. These programs have traditionally involved multiple successive full days of treatment with durations ranging from 5 to over 40 days (Sharp et al., 2017). Feeding problems addressed include food refusal (e.g., Williams et al., 2007), tube weaning (e.g., Sharp et al., 2017), and severe food selectivity (Laud et al., 2009). Additionally, deficits in oral motor functioning, such as lack of chewing (Volkert et al., 2014) or tongue thrust (Gibbons et al., 2007) have been treated in intensive programs. Noel and Silverman (2017) suggest intensive treatment conducted by a multidisciplinary team is the preferred treatment for the elimination of tube feeding due to both its effectiveness and efficiency, especially when compared to outpatient therapy. We assume their comparison is between multidisciplinary intensive treatment, conducted in a setting specifically designed and staffed for the treatment of pediatric feeding problems, to traditional outpatient therapy in which a lone provider, not affiliated with a feeding program, sees the child in the home once weekly, or less. There is a growing number of studies documenting the outcomes of intensive treatment and intensive treatment has also been found to be cost-effective when compared to the expense of prolonged tube feeds (Serban et al., 2020; Williams et al., 2007).

### Outpatient Multidisciplinary Feeding Programs

Alternatively, Ann Davis and her colleagues conducted a pair of studies in which they demonstrated the effectiveness of an outpatient model for tube weaning (Davis et al., 2009, 2016). Their model included appetite manipulation, medications to enhance appetite or control pain, and parent training in behavioral skills. In addition, their model involved multidisciplinary treatment and monitoring which occurred not only in outpatient visits, but with interim phone calls. We do expect the use of similar outpatient interventions will expand to meet the needs of a growing number of children.

### Intensive In-Home Treatment

Intensive treatment in a clinical setting, however, may not be suitable for all children. Even though intensive treatment may save money for the payor, often either an insurance company or state Medicaid program, this form of service delivery could have costs to the family. Lost wages, travel and lodging expenses, insurance co-pays, or other costs incurred during intensive treatment program may make intensive treatment untenable for some families. Studies have described several possible alternatives to intensive treatment, although the number of these studies is limited. Intensive home-based treatment, in which one or more clinicians with expertise in the treatment of pediatric feeding disorders work with the child in the home setting for extended periods of time (T. Taylor, 2020, 2021). While both studies described the treatment of food selectivity, this form of intensive treatment might be applied to other feeding problems. There would seem to be room for growth in the area of intensive in-home treatment, but this approach may be limited by the availability of experienced clinicians and the cost of maintaining staff in the home setting (e.g., travel and lodging).

### Caregiver Training

In a recent study, the prevalence of children with pediatric feeding disorders ranged from 1 in 23 to 1 in 37 children under 5 years of age (Kovacic et al., 2020). For comparison, 1 in 54 children aged 8 years are diagnosed with ASD (Maenner et al., 2020). While the prevalence of feeding disorder is higher than ASD, the number of *behavioral* providers who serve children with ASD is far greater than those who primarily serve children with feeding problems. Even though there is a need for more behavioral providers to work with children with feeding problems, it is not clear that simply increasing the number of these providers is the most efficient method of expanding the number of children served or increasing usage of behavioral interventions. Over the last several years, researchers have examined innovative ways to train caregivers to serve as primary interventionists through various forms of caregiver training procedures (Alaimo et al., 2018; Clark et al., 2020).

Two recent studies have expanded on the use of behavioral skills training (i.e., instructions, rehearsal, modeling, and feedback) to teach caregivers how to implement behavioral interventions to address food selectivity. Clark et al. (2020) evaluated the use of video modeling and instructions to train three parents to implement a structured meal procedure to expand diet variety in children with mild food selectivity. While instructions and video modeling were effective for one parent to achieve a mastery training criterion, two parents still required in vivo prompts and feedback. Alaimo et al. (2018) combined behavioral skills training with general-case training when training three caregivers how to implement a food selectivity intervention. When training caregivers, the authors created scripts to train caregivers how to respond to the commonly document child mealtime responses. After reading the feeding protocol aloud, the experimenters simulated child behavior using one of five scripts in order to provide the caregivers with the opportunity to practice how to respond to a range of child behaviors (e.g., acceptance, expelling, and gagging). Caregivers were then provided feedback on their performance after sampling each script. All caregivers met mastery training criteria of performing at least 90% of the steps of the protocol correctly during an assessment without experimenter feedback. Training was complete within 30 minutes for all three caregivers. Thus, combining behavioral skills training with general case training may be both a comprehensive and efficient way to train caregivers to implement interventions independently without the need for re-training and ongoing feedback across the course of intervention.

### Telehealth Feeding Intervention and Follow-Up

Researchers have also examined the effectiveness of innovative telehealth service delivery models for treatment (Bloomfield et al., 2019) and follow-up services (Peterson et al., 2021). Bloomfield et al. (2019) implemented a food selectivity intervention with a child with ARFID through parent teleconsultation. Using a changing criterion design across foods and food groups, the researchers trained the child’s parent using behavioral skills training via telecommunication to implement a multicomponent feeding intervention. During intervention, the researchers met weekly with the caregiver in order to provide performance feedback and modeling of procedures and instructed the caregiver to continue the intervention for the rest of the week. Results showed an increase in the frequency of bites of non-preferred foods eaten by the child and a high level of parent treatment integrity and consult procedural integrity.

Due to the COVID-19 global health crisis starting in 2020, many clinicians and researchers transitioned to telehealth to treat a range of behaviors. Peterson et al. (2021) conducted a series of studies with children with ARFID who graduated from an intensive outpatient program. Results showed equivalent outcomes along most dimensions measured for those who participated in a follow-up outpatient program in-clinic or via telehealth exclusively.

These studies suggest that telehealth may have great benefits for treating food selectivity and providing follow-up services. Additional research should continue to examine which food selectivity interventions are best suited via telehealth. Future research should also explore if other pediatric feeding disorders (e.g., chewing, packing, and total food refusal) can be effectively treated using a telehealth service delivery model. To our knowledge, the only published behavior analytic feeding literature articles using telehealth interventions have targeted expanding diet variety in children without known oral motor deficits. It is unclear if telehealth interventions are effective and safe for targeting more complex feeding behaviors such as chewing and packing which may stem from oral motor deficits or when treating children who may exhibit severe inappropriate mealtime behavior.

Telehealth might also be used as an extension of existing forms of service provision. For example, telehealth technology could be used during outpatient appointments to allow other family members, school staff, or home-based therapists to participate in the appointment without in-person attendance. Telehealth visits and in-person visits could be alternated not only for the convenience of the family but to allow the service provider to observe the intervention being implemented in the natural setting. While home coaching and consultation remotely is not new, improvements in the technology and the wide-spread adoption of video communications platforms will allow an increased variety of telehealth adaptations.

### The Importance of Evaluating Long-Term Outcomes

While numerous studies have documented the effects of behavioral feeding intervention in the short-term, relatively few studies have documented long-term treatment gains following intervention. In a recent study, Kim et al. (2021) found that treatment gains for the majority of patients receiving intensive multidisciplinary behavioral feeding intervention in a hospital setting can be maintained long term (i.e., at least 1 year following intervention). When examining the length of follow-up for the behavioral feeding interventions reviewed above, it is common for researchers to report follow-up for at most, 3 to 6 months post-intervention. While it can be challenging to obtain long-term follow-up data, it would be beneficial to know if treatment gains following behavioral feeding interventions in various settings (e.g., clinic, home, and school) to treat a range of pediatric feeding disorders can be maintained for up to a year or longer and to determine the portion of children who maintain treatment gains, continue to make improvements, or show regression over time.

It is also important for clinicians to consider how best to fade out treatment components over time and assess how to transition from structured feeding protocols to more naturalistic settings. Without this consideration, some children may remain on structured feeding protocols for much too long, resulting in an overreliance on prompting and reinforcement procedures while other families may opt to fade out treatment protocols too quickly following intervention. A majority of the research focus in our field has been examination of short-term gains in terms of acceptance of novel foods and reductions in inappropriate mealtime behavior. While short-term gains are beneficial, clinicians need to prioritize maintenance and generalization of treatment gains over time. Clinicians should consider developing guidelines for how to assess maintenance of treatment gains over time, train caregivers, program for generalization, and fade out intervention protocols in order to promote long-term treatment gains following behavior analytic feeding interventions.

### Ethical Consideration of Behavior Analytic Feeding Interventions

While ethical issues related to the delivery of behavioral treatment for feeding problems have been well addressed in two recent publications (Fernand et al., 2021; Tereshko et al., 2021), we would like to stress the need for adequate knowledge and training of clinicians who practice in the area of pediatric feeding problems. While a written protocol may provide sufficient information for the implementation of some feeding interventions, many interventions will require hands-on training and supervised practice. For example, a number of studies have described the use of the flipped spoon or Nuk brush (e.g., Volkert et al., 2011). Implementing these interventions in children with chronic food refusal and/or oral motor dysfunction requires training and practice. While still limited, training opportunities are more widely available now than in the past. There are also more opportunities for clinicians to obtain both experience and assistance with intervention development through case collaboration with other clinicians or feeding programs. We earlier described telehealth studies in which clinicians worked with parents remotely. Clinician to clinician teleconsultation is widely used by healthcare providers and could be applied to the area of feeding problems.

Tereshko et al. (2021) made the case for interdisciplinary collaboration with a range of disciplines due to the combined biological and behavioral features of feeding problems. We suggest not only is there a need for interdisciplinary treatment, there is a need for clinicians, to have interdisciplinary knowledge specific to feeding problems. For example, clinicians should know signs of medical conditions that are commonly comorbid with feeding problems such as gastroesophageal reflux and constipation. This is not to suggest behavioral clinicians need to diagnose or treat these or other conditions, but clinicians should know when to alert medical providers to the possible presence of medical issues which may require medical evaluation and/or treatment. For example, in a recent case series, we described three children with scurvy (vitamin C deficiency) who presented with leg pain (Hahn et al., 2020). The first author saw each of the children and based upon reported behavior and the history of oral intake, referred them to medical providers for further testing and all were found to have a deficiency in vitamin C. All three children had been seen by medical providers yet were not diagnosed. Behavioral clinicians will sometimes have the most comprehensive information regarding the child’s intake and feeding behaviors. Additionally, our consistent use of direct observation may make the behavioral clinician the first provider to observe a child’s oral motor dysfunction, including possible aspiration. While it is appropriate not to begin addressing a child’s feeding issues until the child has received medical clearance, this may be insufficient. For many children, identification of medical issues is an on-going process and someone who is having repeated contact with the child over time, the behavior clinician will play an integral part of the healthcare team.

While we are strong proponents of interdisciplinary treatment, we suggest the extent of the interdisciplinary assessment and treatment depends on several factors including the presence of comorbid medical issues, the severity of the feeding problem, and experience of the service provider. Some children present with mild feeding issues, and it may be appropriate for a single provider, behavioral or non-behavioral, to assess and treat the problem with minimal involvement from other disciplines. Other children presenting with more significant feeding problems and/or medical comorbidities will greatly benefit from interdisciplinary treatment as there will likely be numerous factors (e.g., behavioral, oral motor skill deficits, nutritional, and medical) to address as part of intervention.

## Conclusion

As an area of clinical practice, the treatment of pediatric feeding problems has dramatically expanded with more providers using a wider range of interventions to treat a broader array of problems. While many children are treated at a young age, there are also many children who present for treatment much later after they have developed a long history of disordered eating. As services are more widely available, more children will be treated at a younger age. As a larger number of interventions which do not include escape extinction becomes available (e.g., Tereshko et al., 2021), we expect these interventions will be more widely adopted by community providers to help children who have feeding problems, including problems not severe enough for referral to a feeding program or specialist. In this paper, we discussed the current state of the literature regarding food selectivity, packing, and chewing interventions. We also discussed recent approaches to targeting total food refusal and tube weaning. Lastly, we considered several topics valuable to our growth as a field. These included discussing the current range of service delivery models available to families and recent advances in caregiver-implemented and telehealth interventions and evaluating the long-term outcomes of behavior analytic feeding interventions, which still remains an area of weakness in the behavior analytic feeding literature. Lastly, as the number of behavioral providers continues to grow, we must consider best practices for supervision and training for clinicians and trainees in the area of behavior analytic feeding interventions as knowledge of the various factors that may be associated with feeding problems as well as the benefits of interdisciplinary collaboration is key for clinicians pursuing work in this field.
